# Cutaneous ulcers in association with sprue‐like enteropathy secondary to Losartan

**DOI:** 10.1002/ccr3.4645

**Published:** 2021-08-16

**Authors:** Francis Essien, Juakiem Wassem, Joshua Tate, Jared Roberts

**Affiliations:** ^1^ Department of Internal Medicine Keesler Medical Center Keesler Air Force Base Keesler AFB MS USA; ^2^ Department of Gastroenterology Keesler Medical Center Keesler Air Force Base Keesler AFB MS USA; ^3^ Department of Endocrinology Keesler Medical Center Keesler Air Force Base Keesler AFB MS USA; ^4^ Department of Dermatology Keesler Medical Center Keesler Air Force Base Keesler AFB MS USA

**Keywords:** angiotensin II receptor blocker, chronic diarrhea, Losartan, sprue‐like enteropathy

## Abstract

Losartan is an angiotensin II receptor blocker (ARB) which may cause severe sprue‐like enteropathy (SLE) with skin manifestation. Clinicians should be informed of this side effect and its reversibility after cessation of the drug.

## Graphical abstract

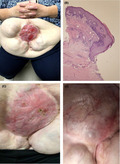

## 1 INTRODUCTION

Angiotensin II receptor blocker‐associated enteropathy is becoming increasingly prevalent within the literature. We report a case of sprue‐like enteropathy associated with Losartan with a unique cutaneous manifestation. Our case of Losartan‐associated enteropathy further suggests that sprue‐like disease may be a class effect of ARBs.

Angiotensin II receptor blocker (ARB)‐induced sprue‐like enteropathy is a rare adverse effect of this class of medication but is increasingly recognized as a potential side effect that clinicians should be aware. The first published cases were from the Mayo Clinic in a case series of Olmesartan‐induced SLE. While reported clinical manifestations of these patients included abdominal pain, severe weight loss, chronic diarrhea, and severe electrolyte derangements, there was no description of cutaneous manifestations. Since that time, only one case of ARB‐induced SLE with cutaneous manifestations has been noted and was associated with Olmesartan, specifically. Symptoms in the above cases resolved within 3–12 months of medication discontinuation. Currently, SLE has yet to be classified as class effect among ARBs; however, there are several cases within the literature that advocate in favor of this idea. We report a case of SLE associated with losartan with a unique cutaneous manifestation.

## 2 CASE REPORT

**FIGURE 1:**
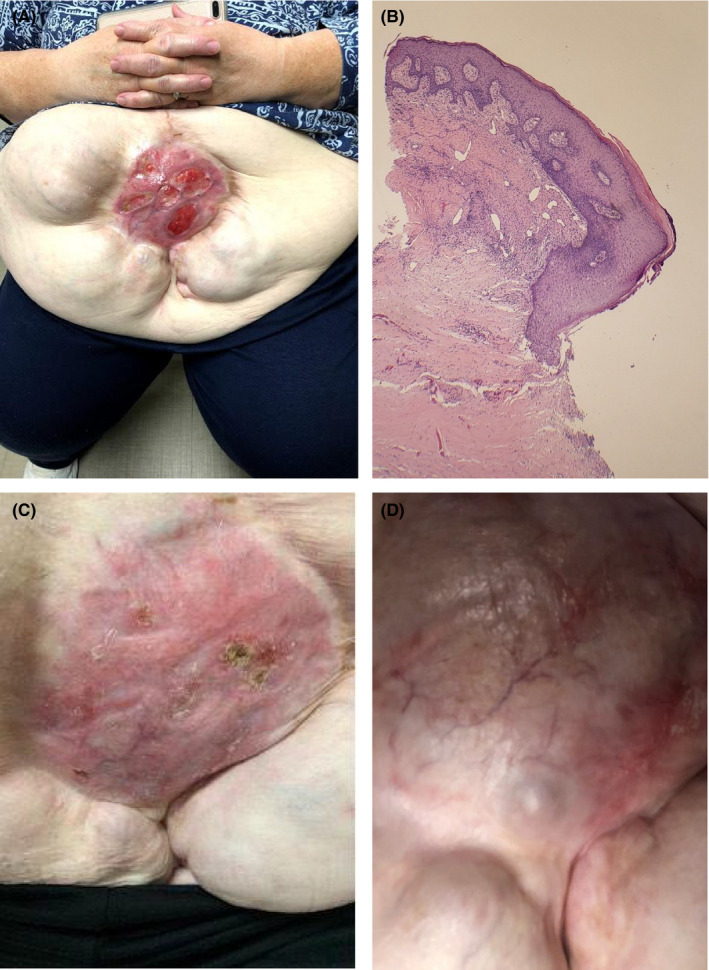
A. 9 × 9 cm scar with multiple ulcerated lesions on the anterior supra‐umbilical region. B. Histopathology: ulceration with extensive dermal fibrosis, chronic inflammation, numerous eosinophils and granulation tissue. C. Supra‐umbilical region 1 month following losartan discontinuation. D. 1‐year follow‐up of supra‐umbilical region—well‐healed graft site without ulceration

**FIGURE 2:**
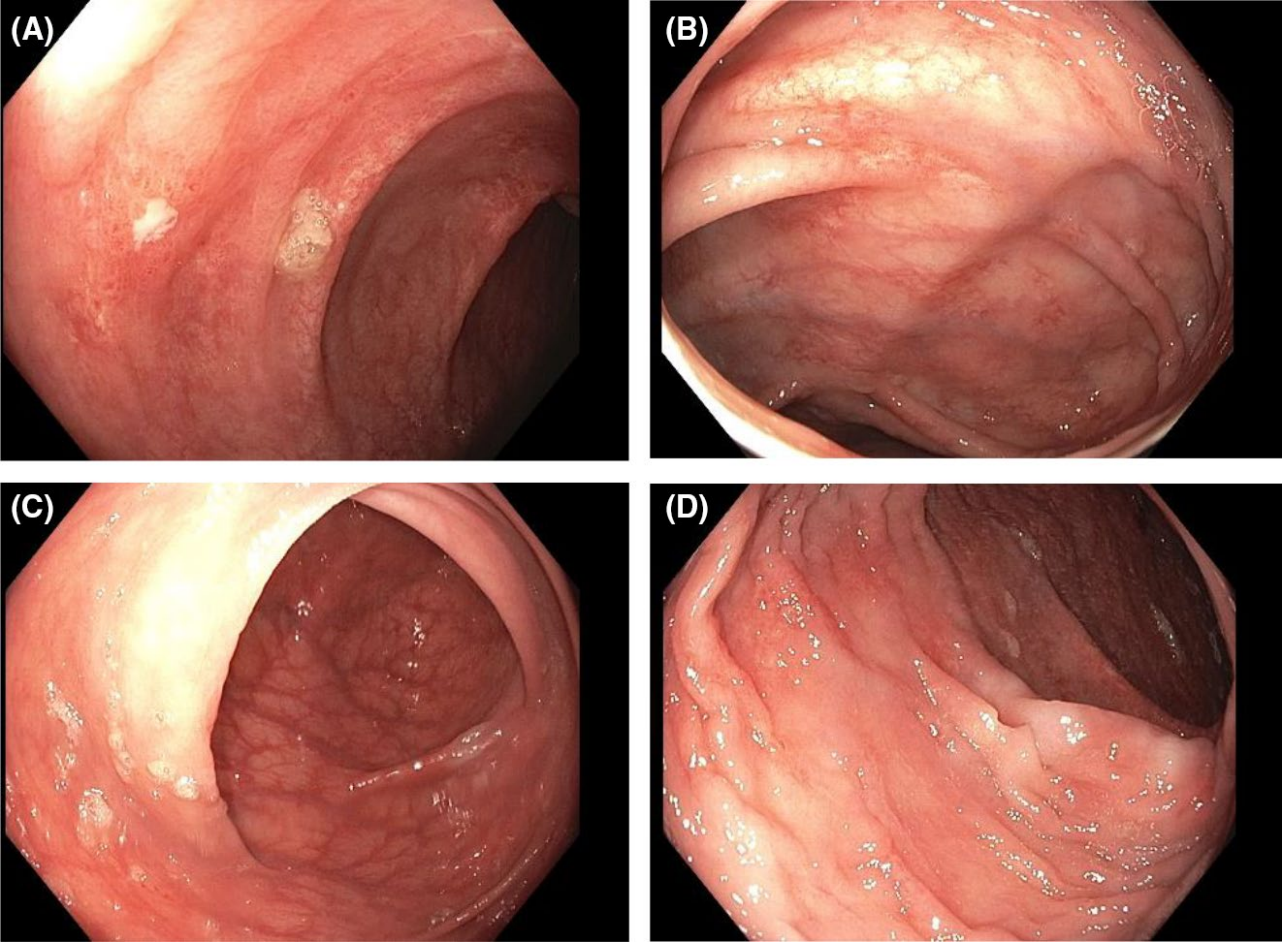
Superficial colonic erosions with surrounding erythema (1A‐arrow). Normal colon mucosa. (1B‐D). Biopsies showing normal colonic mucosa

A 59‐year‐old female patient had an abrupt presentation of diarrhea (>1 month with 5–9 watery stools daily), abdominal discomfort, weight loss, and several large spontaneous ulcerations on the abdomen, limited within scar tissue from a remote bowel perforation surgery (Figure 1A). The combination of diarrhea and ulcerations was concerning for inflammatory bowel disease, pyoderma gangrenosum, or malignancy. Laboratory investigations demonstrated anemia and electrolyte derangements to include hyponatremia, hypocalcemia, hypokalemia, and hypomagnesemia. Chronic diarrhea work‐up was negative, but lactoferrin and calprotectin were elevated (Table 1). A punch biopsy at the edge of an ulcer showed extensive dermal fibrosis with chronic inflammation, numerous eosinophils, and granulation tissue (Figure 1B). PAS and Lu‐5 cytokeratin stains were performed and were unremarkable. The lack of acute or granulomatous inflammation made infection less likely. Otherwise, the classical features of pyoderma gangrenosum, infection, and malignancy were not appreciated. Endoscopy was notable for duodenal villous atrophy, edematous mucosa with scattered area of loss of vascular pattern and ulceration in the proximal colon but histology demonstrated benign colonic mucosa (Figure 2). After months of otherwise unremarkable exhaustive evaluation, there was no clear underlying cause. Of note, the patient had been treated with losartan for more than 10 years for hypertension and given the reports of ARB‐induced sprue‐like enteropathy, losartan was discontinued in October 2019. On follow‐up one month after medication was discontinued, patient noted improvement of the diarrhea and the wounds started healing. Patient followed up with wound care, and over the next several months, the wounds completely resolved as well as her gastrointestinal symptoms. (Figure 1C). One year follow‐up noted complete resolution of the patient's lesions with no recurrence (Figure 1D).

**TABLE 1:** Laboratory results

Thyroid stimulating hormone	1.760	mcIU/ml	(.358–3.74)
Thyroxine free	1.30	ng/dl	(.76–1.46)
ESR	62 (H)	mm/hr	(0–30)
Ferritin	36.6	ng/ml	(8–388)
Fecal fat	Normal <i> <r>		
Fats neutral	Normal <i> <r>		
WBC smear stool	FECES	FEW WBCS SEEN	
OVA & parasites examination panel (x3)	No Ova or parasites found		
Occult Blood Panel	Positive (H)		
Sodium stool	55		
Potassium stool	27	mol/L	
Lactoferrin	147.10 (H) <i> <r>	μg/ml(g)	0.00–7.24
Calprotectin	515 (H) <i> <r>	mcg/g	0–120
Gliadin Ab IgA	6 <i>	Units	0–19
Gliadin Ab IgG	2 <i>	Units	0–19
Tissue Transglutaminase Ab IgA	<2 <i>	U/ml	0–3
Tissue transglutaminase Ab IgG	<2 <i>	U/ml	0–5
Endomysial Ab IgA	Negative		Negative
Vasoactive intestinal peptide	<16.8 <i>	pg/ml	0.0–58.8
Elastase pancreatic	234 <i>	Microgram elastase/gram	>200
Somatostatin	25 <i>	pg/ml	Adult <or=30
5‐Hydroxyindoleacetate	5.0	mg/24 Hr	0.0–14.9
Gastrin	20 <i>	pg/ml	0–115
Stool culture	Scant Heavy Growth of Normal Flora 72 hours		
Clostridium difficile DNA	Negative <i>		
027/NAP1 Strain	Negative		
Campylobacter PCR	Not detected		
C Difficile Toxin A/B DNA	Not detected		

## 3 DISCUSSION

The increasing armamentarium of pharmacological agents has resulted in a multifactorial fusillade of effects within the gastrointestinal and cutaneous systems. The small intestine is the most common site of adverse drug reaction due to the delicate balance between the enteric and central nervous system.^1^ Often there is a temporal relationship between the drug ingestion and onset of symptoms, but this can sometimes manifest inconsistently and unpredictably.^1^, ^2^ Currently, there is no universally accepted definition or classification for drug‐induced adverse effects due to the inability to properly delineate the complex mechanism of action due to complexity and combination of effects.^1^ Marietta et al proposed a diagnostic criteria of olmesartan‐associated enteropathy (OME) to include chronic diarrhea greater than 4 weeks while taking Olmesartan Medoxomil, alternate cause unable to be established after a systematic diagnostic evaluation for disorders associated with non‐responsive celiac disease and clinical improvement after discontinuation.^2^ However, diagnosis and management are largely driven by laboratory, endoscopic and/or imaging findings.

Very few cases of ARB‐induced sprue‐like enteropathy, other than olmesartan, have been reported.^1^, ^2^ There has only been one reported case with cutaneous manifestations and ARB‐induced sprue‐like enteropathy. In 2016, Hammoudi et al published the first case of olmesartan‐induced enteropathy with “papulo‐erythematous lesions and scabs” with full resolution within a week following cessation of the medication.^3^ In 2012, an association between Olmesartan and SLE was first noted by Rubio‐Tapia et al and this was further propagated with the publication of a case series in 72 patients with villous atrophy and seronegative celiac disease.^3^ Nielsen et al published a case series of patients with collagenous sprue characterized by marked villous blunting, intraepithelial lymphocytes, and thickened sub‐epithelial collagen secondary to Olmesartan and complete resolution following cessation of the medication.^4^ Uehara et al. recently reported a Olmesartan‐induced sprue‐like enteropathy manifesting as Wernicke‐Korsakoff syndrome secondary to severe malabsorption.^5^ The majority of case reports have been associated with Olmesartan medoxomil, the prodrug formulation, but numerous case reports increasingly postulate that SLE may be a class effect. Alzueta et al. demonstrated a case of Telmisartan associated enteropathy with cessation following drug withdrawal.^6^ DeBortoli at al in 2017 conducted a large based population study on patients treated with ARB therapy in Italy and Germany which associated ARB‐induced SLE but with a relatively low incidence.^2^, ^7^ Hence, it can be postulated that this is most likely a class effect.

The pathophysiology of angiotensin‐related sprue‐like enteropathy is currently unknown at this time but there are several theories primarily driven by Olmesartan since this is the most common medication in the literature. The renin‐angiotensin‐aldosterone system plays an important role in the regulation of electrolytes, primarily sodium and water secretion within the jejunum and colon.^2^, ^8^ It has been postulated that within certain predisposed patients this mechanism may be more strongly activated resulted due to increased bioavailability of angiotensin II receptors.^9^, ^10^ Several studies have demonstrated that many of the common pathogenic pathways seen in celiac disease are also present within that of ARB‐induced sprue‐like enteropathy.^10^, ^11^ There is an increased number of CD8+‐positive T cells and granzyme‐positive B cells resulting in destruction of the lamina propria and epithelial layer.^2^, ^10^, ^12^ Previous studies have demonstrated an increased number of CD8+‐positive T cells which express interleukin 15 and 15R resulting in the disruption of tight junction complexes such as in refractory celiac sprue.^10^, ^12^ Given some of the known histopathology overlap observed in celiac disease and ARB‐induced sprue‐like enteropathy, a cutaneous manifestation is not unexpected, although the cutaneous manifestations may differ.^13^, ^14^ Celiac disease is associated with dermatitis herpetiformis whereas ARB‐induced sprue enteropathy may cause ulcerative lesions.^14^

## 4 CONCLUSION

The pathophysiology of ARB‐induced sprue‐like enteropathy is currently unknown with even less understood regarding the connection to a cutaneous manifestation. However, the punch biopsy and duodenal samples did demonstrate findings consistent with drug eruption, and after months of symptoms, a few hospitalizations, and several failed therapies, the treatment that ended up improving her condition was discontinuation of the losartan. Our case of losartan induced enteropathy further suggests that sprue‐like enteropathy with cutaneous manifestations may be a unique but rare class effect of ARBs and should be considered when evaluating patients with similar clinical findings.

## CONFLICT OF INTEREST

The authors declare that they have no competing interests.

## AUTHOR CONTRIBUTIONS

Study conception and design: Francis Essien D.O., Wassem Juakiem M.D., Jared Roberts M.D. Data collection: Francis Essien D.O. Analysis and interpretation of results: Francis Essien D.O., Jared Roberts M.D. Draft manuscript preparation: Francis Essien D.O., Joshua Tate M.D.

## ETHICAL APPROVAL

No ethics board approval was needed for this case report. Consent was obtained from the patient to publish the case.

## Data Availability

Data sharing is not applicable to this article as no new data were created or analyzed in this study.

## References

[1] Scarpignato C, Bjarnason I. Drug‐induced small bowel injury: a challenging and often forgotten clinical condition. Curr Gastroenterol Rep. 2019;21:55. 31720893 10.1007/s11894-019-0726-1

[2] Marietta E, Nadeau A, Cartee A, et al. Immunopathogenesis of olmesartan‐associated enteropathy. Aliment Pharmacol Ther. 2015;42:1303‐1314. 26423313 10.1111/apt.13413 PMC4626300

[3] Hammoudi N, Dior M, Giraud V, Coffin B. Olmesartan‐induced Enteropathy, associated with cutaneous lesions. Clin Case Rep. 2016;4:379‐382. 27099732 10.1002/ccr3.531 PMC4831388

[4] Nielsen J, Stephen A, Lewin M. Angiotensin‐II inhibitor (Olmesartan)‐induced collagenous sprue with resolution following discontinuation of drug. World J Gastroenterol. 2013;19(40):6928‐6930. 24187471 10.3748/wjg.v19.i40.6928 PMC3812495

[5] Uehara T, Ikusaka M, Ohira Y, et al. Olmesartan‐induced enteropathy manifesting as Wernicke‐Korsakoff syndrome. Int Med. 2016;55:3675‐3678. 10.2169/internalmedicine.55.7388 PMC5283972 27980272

[6] Alzueta N, Echeverria A, Sanz L, Fontela AT, Montenegro L, Garjon J. Telmisartan‐induced sprue‐like enteropathy: a case report. Eur J Hosp Pharmacy. 2020;27:49‐51. 10.1136/ejhpharm-2018-001669 PMC6992972 32064089

[7] Shahzad MA, Harding D, Ruszkiewicz A, Tran E, England G, Philpott H. Gastrointestinal: Olmesartan‐induced Enteropathy. J Gastroenterol Hepatol. 2018;33:1691. 29968297 10.1111/jgh.14317

[8] Sher M, Murray M, Mcguire L, Fitzpatrick S, Kurtkoti J. Olmesartan‐induced Enteropathy: A Rare Side Effect of a Common Medication. Cureus. 2019;11(12):e6400. 31970030 10.7759/cureus.6400 PMC6964959

[9] Bolognia J, Jorizzo J, Schaffer J. Dermatology, 4th edn. Amsterdam: Elsevier Saunders; 2012.

[10] Reunala T, Salmi TT, Hervonen K, Kaukinen K, Collin P. Dermatitis herpetiformis: a common extraintestinal manifestation of coeliac disease. Nutrients. 2018;10:602. 10.3390/nu10050602 PMC5986482 29757210

[11] Melis C, Struyve M, Steelandt T, Neuville B, Deraedt K. Sprue‐like enteropathy, do not forget olmesartan! Dig Liver Dis. 2018;50:621‐624. 29625908 10.1016/j.dld.2018.03.017

[12] Van Gils T, Robijn RJ, Bouma G, Neefjes‐Borst EA, Mulder CJJ. A Pitfall in Suspected (refractory) Celiac Disease: Losartan‐Induced Enteropathy. Am J Gastroenterol. 2017;112:1754‐1755. 29109495 10.1038/ajg.2017.281

[13] Negro A, Rossi GM, Santi R, Iori V, De Marco L. A Case of Severe Sprue‐like Enteropathy Associated with Losartan. J Clin Gastroenterol. 2015;49:794. 10.1097/MCG.0000000000000383 26166143

[14] Kamal A, Fain C, Park A, et al. Angiotensin II receptor blockers and gastrointestinal adverse events of resembling sprue‐like enteropathy: a systematic review. Gastroenterol Rep (Oxford). 2019;7:162‐167. 10.1093/gastro/goz019 PMC6573796 31217979

